# Lengthy Shifts and Decision Fatigue in Out‐of‐Hours Primary Care: A Qualitative Study

**DOI:** 10.1111/jep.70050

**Published:** 2025-03-13

**Authors:** Mona Maier, Louisa Lawrie, Daniel Powell, Peter Murchie, Julia L. Allan

**Affiliations:** ^1^ Health Psychology, Institute of Applied Health Sciences University of Aberdeen Aberdeen Scotland; ^2^ Academic Primary Care, Institute of Applied Health Sciences University of Aberdeen Aberdeen Scotland; ^3^ Division of Psychology, Faculty of Natural Sciences University of Stirling Stirling Scotland

**Keywords:** clinical decision‐making, decision fatigue, general practitioners, interview, medical decision‐making, nurses

## Abstract

**Rationale:**

Demands on healthcare workers are high: services are stretched, shifts are long and healthcare professionals (HCPs) regularly work lengthy periods without a break. Spending time continuously ‘on task’ changes decision‐making in predictable ways, as described by the ‘decision fatigue’ phenomenon where decision‐makers progressively shift towards making less cognitively effortful decisions as the time worked without a break increases. This phenomenon has been observed repeatedly in large quantitative observational studies, however, individual healthcare workers' experiences have not been explored.

**Aims:**

This qualitative study aimed to explore general practitioners' (GPs) and advanced nurse practitioners' (ANPs) experiences of working for lengthy periods in an out‐of‐hours primary care service in the UK. This included exploration of self‐perceived changes in decision‐making throughout a work shift, and mitigating strategies used to avoid changes in decision‐making over time.

**Design:**

Semi‐structured interviews were conducted online. An inductive thematic analysis was carried out to identify salient issues articulated by participants.

**Setting and Participants:**

The interview sample (*n* = 10) comprised ANPs (*n* = 5) and GPs (*n* = 5) who regularly worked within the out‐of‐hours primary care service across a regional National Health Service (NHS) health board.

**Results:**

HCPs (GPs and ANPs) provided insights into their experiences during lengthy shifts and the impact of prolonged periods of work on clinical decision‐making. Four main themes were identified and developed: (1) HCPs are aware of decision fatigue effects over the course of a shift; (2) Multiple factors help and hinder stable decision‐making quality; (3) HCPs deliberately use strategies to help keep the quality of their decision‐making stable; and (4) HCPs are aware of contextual changes, likely related to the decision fatigue phenomenon.

**Conclusions:**

The findings of this study underscore the intricate interplay of personal, social and systemic factors in decision‐making quality and highlight HCPs' deliberate efforts to mitigate decision fatigue's effects in practice.

## 1 Introduction

Healthcare professionals (HCPs), particularly those in primary care, frequently work without being able to take adequate breaks. Recent surveys have indicated that UK general practitioners (GPs) are facing unprecedented workload demands [1], and a significant majority (77%) do not take any rest breaks during clinic sessions [2]. Similarly, nurses working in general practice have reported feeling overworked, perceiving workload as being higher than it was before the COVID‐19 pandemic [3]. This is despite strategies to improve matters, such as UK minimum rest requirements, which stipulate an entitlement to a minimum 20‐min rest break where the working day is longer than 6 h [4, 5]. Engaging in continuous and unbroken periods of work in this manner provides an opportune environment for the development of ‘decision fatigue’. Decision fatigue refers to the gradual tendency to lean towards making mentally easier choices as the duration of uninterrupted work increases [6]. In the primary care context, decision fatigue has likely ramifications for consistent and equitable patient care, patient and staff well‐being, and the job satisfaction of HCPs.

Decision fatigue has been observed across a range of contexts [7, 8, 9], and numerous studies reveal compelling insights into the impact of decision fatigue on HCPs. Nurses exhibit an increased likelihood of making more conservative triaging decisions (i.e., arranging for callers to see another health professional the same day as opposed to deferring appointments to a later date) as the time since their last break accumulates [10]. Hand hygiene compliance rates in hospitals reduce significantly from the beginning to the end of a typical 12‐h work shift [11]. Surgeons become less inclined to recommend surgical interventions towards the end of their shift [12]. GPs administer a reduced number of flu vaccinations [13] and exhibit a decreased tendency to request cancer screenings for their patients as the clinic sessions progress [14]. GPs also become more likely to prescribe antibiotics and opioids as the working day progresses [15, 16].

Existing decision fatigue studies in the healthcare context are mostly quantitative and observational. No qualitative study has specifically explored health professionals' subjective experience of decision fatigue. Consequently, there is a lack of understanding about whether HCPs are consciously aware of time and fatigue‐related changes in their decision‐making when working lengthy periods. Some studies suggest that HCPs are not consciously aware of when decision fatigue impacts them [6]. However, published expert opinion pieces and editorials [17, 18, 19, 20, 21, 22] suggest anecdotal awareness of the phenomenon amongst health professionals. While HCPs might not have heard of the term ‘decision fatigue’ per se, they regularly reflect on their practice as part of professional appraisals and, therefore, may have become aware of patterns in their own decision‐making [23].

Due to the direct effects on care decisions and potential negative impacts on workforce satisfaction, a deeper understanding of HCPs' experiences of decision fatigue is required. This is the first qualitative study which aims to uncover and understand GPs' and advanced nurse practitioners' (ANPs) experiences of working lengthy shifts and clinical decision‐making throughout these shifts in an out‐of‐hours primary care service.

## 2 Methods

### 2.1 Study Design

This qualitative study employed semi‐structured interviews. The interview topic‐guide was designed to explore general experiences of working lengthy shifts and perceived changes in clinical decision‐making throughout a shift.

The interview guide was developed by researchers well‐versed in decision fatigue (M. M., J. A., D. P.), piloted with two individuals with experience working in out‐of‐hours primary care, and refined to suit common job roles, employment patterns (full‐time, part‐time or bank staff), and working arrangements (advice calls, centre consults and home visits). The interview guide was iteratively updated as the interviews progressed.

### 2.2 Setting

The study setting is an urgent primary care service serving a large regional health board in Scotland and catering to patients who cannot wait until regular hours to see their GP. The service is accessed either via referral from NHS24, a Scotland‐wide telephone helpline or by walk‐ins or referrals from Minor Injury Units. The service covers a regional population of 586,530 individuals [24], operating from 18:00 to 08:00 on weekdays and 24 h on Saturdays, Sundays and public holidays.

The eight service bases are staffed with ANPs, GPs, Paramedic Practitioners, and Drivers who all support clinical operations. GPs and ANPs are the HCPs who directly manage patients' healthcare needs with the support of the other staff. Both professional groups have overlapping tasks and responsibilities. ANPs have slightly fewer accountabilities (e.g., they do not deal with mental health issues) and are provided with additional access to telephone support from GPs. ANPs and GPs may assume various staff contracts, such as full‐time, part‐time or bank staff employment. Bank staff members have the flexibility to choose available shifts on a weekly basis.

In line with other out‐of‐hours primary care services across the country, the service offers patients advice and/or assessment via telephone advice/consultation, in‐person consultation at the nearest centre, or a home visit as necessary.

### 2.3 Participants/Sampling

Study participants were GPs or ANPs working regularly within the regional urgent primary care service. An even balance between ANP and GP participants was sought to ensure equal representation of both occupational groups. The interview advert (including the consent form and participant information sheet) was distributed to all service staff by the service manager and posted on social media.

### 2.4 Data Collection

Interviews were conducted between April and June 2023 by M. M. via Microsoft Teams [25] and recorded with the built‐in recording function. Recruitment continued until thematic saturation was reached, that is, no new codes or themes emerged [26]. The interviews were transcribed verbatim and anonymised (M. M.). All participants provided written and verbal informed consent. Ethics approval was granted by the East of England – Cambridge East Research Ethics Committee in October 2022 (REC reference 22/EE/0259).

### 2.5 Data Analysis

A thematic inductive approach to analysis was adopted to explore the broader contextual experiences of working for extended periods and perceived changes in clinical decision‐making over the work shift [27]. Following a careful review and refinement of the identified themes, a thematic framework was developed by M. M., overseen and reviewed by L. L. and J. A. This framework was further refined through group discussions between M. M., L. L., D. P. and J. A.

To ensure the accuracy and consistency of the coding process, a double coder (L. L.) examined the themes and descriptions of participants' responses across three transcripts. Any instances of coding discrepancies were subjected to thorough discussion between M. M. and L. L. to achieve consensus and maintain the integrity of the data analysis. Data analysis was supported using NVivo (version 12) [28] and group discussions for theme refinement were supported using the Miro App, a software that enables digital visualisations of mind mapping [29].

## 3 Results

### 3.1 Demographics

Ten out‐of‐hours primary care clinicians (five GPs, five ANPs) participated in interviews lasting 25–45 min. As shown in Table 1, participants varied in terms of their professional experience and the frequency with which they worked out‐of‐hours shifts. In addition to their clinical roles within the service, four participants held supervisory and/or leadership positions.

**Table 1:** Demographic data for the interviewees.

Participant	Job role	Years of experience	Shift frequency
P1	ANP	5–10 years	5–10 per month
P2	ANP	> 10 years	> 10 per month
P3	ANP	< 5 years	> 10 per month
P4	ANP	< 5 years	> 10 per month
P5	ANP	5–10 years	> 10 per month
P6	GP	> 10 years	5–10 per month
P7	GP	< 5 years	< 5 per month
P8	GP	5–10 years	5–10 per month
P9	GP	> 10 years	> 10 per month
P10	GP	> 10 years	> 10 per month

### 3.2 Findings

Participants raised several key issues concerning their experiences of working lengthy periods, reflecting that: (1) HCPs are aware of decision fatigue effects over the course of a shift, (2) Multiple factors help and hinder stable decision‐making quality, (3) HCPs deliberately use strategies to help keep the quality of their decision‐making stable, and (4) HCPs are aware of contextual changes, likely related to the decision fatigue phenomenon. The subsequent sections delve into a comprehensive analysis of these issues.

Theme 1 HCPs are aware of decision fatigue effects over the course of a shift.

Participants reported that they experience changes in their decision‐making process and associated behaviours over the course of their shift. In some cases, this was described as a general phenomenon (Theme 1.1), other times participants specifically referred to certain types of decisions or associated behaviours that change over the shift (Theme 1.2). Two participants insisted that their decision‐making remains completely consistent over time, one of which caveated this by attributing the consistency to only working shorter shifts.

3.2.1

1.1 *General changes to the decision‐making process.*

Most participants described that the decision‐making process becomes more challenging towards the end of the shift. Specifically, HCPs became more doubtful about their own decisions as they progressed to later stages within the shift or made decisions more quickly.

I think, by default, the decision‐making becomes more challenging as the day goes on […] just because you've had hours of critical thinking.

P2, ANP

I start to see zebras^1^ [later in the shift] or panic that I'm missing zebras. Whereas I feel much more comfortable in my clinical decision‐making earlier in the day.

P1, ANP

1.2 *Specific changes in decision‐making and associated behaviour.*

Perceived changes in decision‐making were also specifically tied to associated behaviours. This included an increased likelihood of prescribing antibiotics/benzodiazepines at later stages of the shift, finding it more difficult to admit a patient to hospital later in a shift (when hospital bed capacity has been noted as scarce), and being more hesitant to take an observe and wait approach. Furthermore, some participants noted that the quality of their documentation into medical records decreases, their productivity (the number of patients seen within an hour) decreases, and articulating things clearly becomes more challenging.

I think you do get a bit of “ugh you just want the antibiotics, whatever, here you go”. […] whereas earlier in the shift I might have felt I had the strength to say [to the patient] “actually this this sounds like you have an upper respiratory tract infection, antibiotics are not appropriate in those cases”.

P1, ANP

At the end of this shift, […] I'm more likely to escalate^2^ because I feel more worried about things. It probably will affect my prescribing as well because I'll be more worried that I'm missing things.

P5, ANP

I would say that if I'm tired, I'm perhaps more likely to say, “oh, well, I'll send round half a dozen diazepam^3^”.

P6, GP

3.2.2

Theme 2 Multiple factors help and hinder stable decision‐making quality.

Interviewees described a multitude of factors that were perceived to prevent or support consistent decision‐making. These were often double‐edged, acting as either barriers or facilitators to consistent decision‐making depending on context. Factors identified under this theme were grouped into individual factors (Theme 2.1) and environmental factors (Theme 2.2).

2.1 *Individual factors*

2.1.1 Physiological factors

3.2.3

3.2.3.1

Interviewees stated that physiological factors act as barriers to consistent decision‐making. Getting hungry towards lunchtime and feeling tired during night shifts impacted decision‐making. Feeling hungry was associated with rushing decisions to finish a patient consultation before finding time to have lunch. Feeling tired during night shifts was discussed by most interviewees and was perceived to make consistent decision‐making difficult.

Towards the end, I would definitely say hunger strikes because of lunchtime and […] then [you're] just trying to get everything done. You're a […] little bit quicker to try and get away on time.

P7, GP

It's different challenges; night shift it's because of the time of day and your brain itself. You're fighting against your body wanting to go to sleep.

P5, ANP

2.1.2 Identity

3.2.3.2

Interviewees credited their identity as a facilitator for stable decision‐making quality. They linked their professionalism, personality and experience to supporting them in keeping decision‐making quality stable throughout a shift. Years of professional training, years of experience working within the service, having a structured approach to consultations and a conscientious personality were linked to consistent decision‐making. Professionalism was predominately discussed by GPs though one ANP also referred to this.

I'm very formulaic […] I'm very structured […] maybe quite rigid in the way […] it's almost autistic, it's very sort of […] for me it's very black and white. You know what I do, it's very rigid.

P6, GP

I don't think my decision‐making process really changes that much because […] I'm a cautious person.

P7, GP

GPs are probably the same, but nurses definitely it's instilled in us from our NMC [Nursing and Midwifery Council – regulator for nursing and midwifery professions in the UK]. So, our professional guidelines is that you've got to make the best for every patient and I think it is instilled in us.

P5, ANP

2.2 *Environmental factors*

2.2.1 Workload

3.2.4

3.2.4.1

Workload‐related aspects identified as barriers to stable decision‐making quality included long waiting lists, length and difficulty of prior consultations, additional supervisory tasks and staff shortages.

If you spend a long time with one patient beforehand it […] can affect the next patient.

P7, GP

One of the factors increasing the stress is the number of patients. Usually at the beginning of the shifts, the patients are less […]. At the end of the shift, I think because everybody is going a little bit slower, [… the] number of patients waiting increases. I think that puts more pressure and stress on the service.

P10, GP

2.2.2 Social

3.2.4.2

Interviewees emphasised social aspects as both barriers and facilitators to stable decision‐making quality. This included social norms and social support between colleagues, patient expectations and patient feedback. There was a norm described, where practitioners were reluctant to pass on work to those colleagues taking over, and it typically involved speeding work up towards the end of the shift to ‘deal with’ as many patients as possible. Receiving social support from colleagues throughout the work shift as well as positive interactions with patients (expressing gratitude) positively impacted motivation and morale. However, patient expectations were often described as differing from clinician expectations, which could create conflict, putting uncomfortable pressure on the clinician.

I hate the idea that I'm passing work on to somebody, even if I know […] it's the right thing to do. […At] the end of the shift, there's that pressure of trying to get it as tidy as it can be for the person that's coming on after you.

P2, ANP

Whether it's a GP colleague [or] an ANP colleague, I always find that sharing something does help.

P6, GP

If I feel like I'm […] isolated and there's lots of the team not working hard, that makes my fatigue and [sense of being] overwhelm[‐ed] worse, definitely.

P1, ANP

People [patients] know how to manipulate […]. They insisted they want antibiotics, they want steroids, they want this and that, because that's the only thing that will help them. A few of them, I have reluctantly given it to them. Yeah, because what's the point of having a fight with them? […] they're never gonna give up.

P3, ANP

2.2.3 System‐related

3.2.4.3

Several system‐related barriers and facilitators were identified including general factors such as lack of spaces in hospitals which make admission decisions logistically difficult, and service system‐specific factors that do not compare to other settings. One interviewee noted that the decisions to be made during out‐of‐hours shifts do not vary as much as in other settings and are, therefore, less taxing because one can anticipate most of the decisions that need to be made within the smaller set of reasons for consulting. A barrier to stable decision‐making specific to the service setting seemed to be the added task of selecting which patient to speak to next. In the service computer system, all clinicians have access to the full list of patients waiting to be seen across the region. Clinicians must select patients from the list based on urgency criteria and geographical location.

During your shift, you'll get an alert from the hospital saying please do not admit patients. And that can be very hard […] that that can affect your decision.

P5, ANP

I think it is probably different in out‐of‐hours because there's only a couple of really big decisions, […] whereas in daytime general practice is maybe even more decisions […]. The nice thing with out‐of‐hours is that it's not as pressured as probably daytime general practice is […]. There's lots of people working in the centre, so therefore it's not your whole responsibility to be seeing the next patient.

P7, GP

Theme 3 HCPs deliberately use strategies to help keep the quality of their decision‐making stable.

All interviewees revealed strategies they use to keep their decision‐making consistent across the work shift. Strategies were clustered into demand reduction strategies (Theme 3.1) and demand management strategies (Theme 3.2).

3.1 *Demand reduction*

3.2.5

Strategies used to reduce demand and therefore reduce the potential for rising decision fatigue included taking breaks (Theme 3.1.1) and altering the usual shift pattern (environmental self‐regulation; Theme 3.1.2).

3.1.1 Breaks

3.2.5.1

Taking breaks was a strategy discussed by all participants and was generally seen as effective in alleviating decision fatigue effects. However, the practicality of taking breaks was questioned by some who felt they were not able to take breaks, or that taking breaks is sometimes counterproductive.

If you take your break, you feel that again there's a sharpness at your work. You can probably do a bit more work for the next hour or so.

P8, GP

If you take a break, you're almost harming yourself because you've maybe got away, but then you've just made the work accumulate more.

P5, ANP

The way breaks were spent varied from looking at one's phone, going on walks, meditating for 30 s, to having a cup of tea/coffee while chatting with colleagues.

If that overwhelm is quite bad, I will sort of get up from the office I'm in if I'm doing telephone calls, and I will go for a little bit of a walk around the building.

P1, ANP

3.1.2 Environmental self‐regulation

3.2.5.2

Strategies that alter the usual shift pattern and thereby reduce overall demand were specific to individual interviewees. One interviewee's strategy was to only work shorter shifts as longer shifts are too taxing. Other interviewees choose to only work daytime shifts or to only work a few out‐of‐hours shifts per month.

I tend to do days now. I'm getting older and part of the reason I moved to daytime primary care was I struggle with night shifts.

P1, ANP

3.2 *Demand management*

3.2.6

Demand management strategies included cognitive self‐regulation (Theme 3.2.1), routine approaches to tasks (Theme 3.2.2), and behavioural self‐regulation (Theme 3.2.3). Rather than finding ways to reduce the externally imposed demand to keep decision‐making quality stable, these strategies focus on finding new ways to match demands that are otherwise beyond HCP's initial capacity.

3.2.1 Cognitive self‐regulation

3.2.6.1

Participants described the use of self‐directed regulation to make a decision and actively expending additional cognitive effort before coming to a decision.

The more tired I become, the harder I try to make the right decision. I consciously try to step up, if you like.

P6, GP

3.2.2 Routine approaches to task

3.2.6.2

Participants also described doing certain things in a routinised way; routinely seeing every patient as unique, eliminating distractions and task‐switching. This means that certain approaches are habituated and therefore require less cognitive effort to enact.

I regard every patient‐contact as being unique and […] I try to be the same with everybody.

P6, GP

I always start each case as fresh […]. I've got a structure that I do. So my triaging is my structure whether I'm doing it face to face, whether someone else has triaged them, I still just stick to my structure and what I'm wanting to do.

P5, ANP

3.2.3 Behavioural self‐regulation

3.2.6.3

Additional use of guidelines and tools, cherry‐picking of patients, social support and advice seeking were behavioural self‐regulation strategies used to deal with increasing demand levels over the course of a shift.

Additional use of guidelines and tools was used mainly to confirm pre‐formed decisions and alleviate increasing levels of doubt over the course of the shift.

Even though it's the 11th hour of your shift, you want to be treating the person the same, as if it was the first hour […]. I would check the NICE guidelines three times more, even when I already pretty much know what it says.

P5, ANP

Cherry‐picking of patients was a strategy that was discussed by most interviewees, either as something they do themselves or something that they perceive others do. Cherry‐picking meant that HCPs actively selected the next patient based on criteria of ease (hoping that the consultation will be less effortful), as opposed to recommended selection criteria such as urgency or longest wait time. Some interviewees described cherry‐picking as a viable strategy, others said they used it, but it could often turn out to be a fallacy as patients might look ‘easy’ on paper but then turn out to be more complex. Others discussed cherry‐picking as something their peers do that causes problems for them personally or for the service.

It's something that really annoys me. I find it [cherry‐picking] really frustrating because my experience is that GPs will do that. […] I try very, very hard actually not to do that […] I don't default to that. And actually, that is part of the issue that then overwhelms me.

P1, ANP

I must admit, […] coming to the end [of the shift] I might […] not pick up [patients with] abdominal pain, only because I know it could be a multitude of things and I'd be so worried I'd miss something.

P4, ANP

Social support and advice‐seeking were discussed widely as a strategy to support consistent decision‐making. Some interviewees spoke about seeking advice as a spontaneous action that occurs naturally, while others referred to it in the context that the service provides a supervisor for each shift who can be asked for advice.

I can just go and speak to one of my colleagues. […] you know, run this past [them] just to make sure I'm making the right [decision].

P3, ANP

There was some variety between different service centre locations, as some were staffed by one person who needed to pick up the phone and actively contact their peers to ask for advice. Other centres were staffed by bigger groups with more opportunities for casual social support and advice‐seeking. One interviewee specifically stated that they do not seek advice from their colleagues.

Theme 4 HCPs are aware of contextual changes, likely related to the decision fatigue phenomenon.

In addition to the awareness of decision fatigue effects on decision‐making throughout a shift, participants discussed several emotional and cognitive changes within themselves that are likely related to the decision fatigue phenomenon.

Changes in emotions were perceived widely. This included declining compassion and empathy towards patients over time. Interviewees stated their level of empathy towards patients can wane over the course of the shift which leads to asking fewer questions to the patient. It also increased the potential for conflict and for conversations to take a discourteous tone.

My style changes as the shift goes on […] I'm described as an empathic Doctor; […] towards the end of some brutal […] shift my empathy is minimised.

P9, GP

Participants also highlighted cognitive changes over a shift in areas such as decreasing motivation, feeling in control, capacity, confidence and increasing cautiousness. Cautiousness was discussed exclusively by ANPs who described they become overly suspicious of symptoms.

The fatigue makes me feel, “ugh I don't want to be here, I want to go home”.

P3, ANP

As time goes on […] I almost question myself, […] are you just tired and missing things, […] is it your tiredness just saying this or do you do you [actually think this is right]?

P4, ANP

## 4 Discussion

The GPs and ANPs shared insights into their experiences of working lengthy shifts and the impact on clinical decision‐making throughout their shifts. Four main themes were constructed from the interview data. First, HCPs acknowledged the impact of decision fatigue on their decision‐making processes throughout a shift, noting a general decline in decision quality and systematic changes in specific types of decisions, such as prescribing antibiotics and benzodiazepines more frequently. Second, various factors influenced decision‐making consistency, including physiological states (e.g., hunger, fatigue), professional identity, workload, social dynamics and systemic issues. For instance, feeling tired or hungry negatively affected decisions, while professionalism and experience were perceived as stabilising factors. These factors are not directly linked to decision fatigue but give a broader insight into what might impact consistent decision‐making. Third, HCPs employed strategies to maintain decision quality, categorised into demand reduction (e.g., taking breaks, adjusting shift patterns) and demand management (e.g., cognitive self‐regulation, routine approaches, behavioural self‐regulation like using guidelines and seeking social support). Lastly, HCPs reported emotional and cognitive changes likely linked to decision fatigue, such as decreased empathy, motivation and confidence, along with increased cautiousness and potential for conflict. These findings highlight the complex interplay of personal, social and systemic factors in maintaining decision‐making quality and the conscious efforts by HCPs to mitigate the effects of decision fatigue in their practice.

The systematic changes in specific types of clinical decisions, such as prescribing antibiotics and benzodiazepines, partly align with existing quantitative observational studies that explore the effects of decision fatigue on clinical decision‐making. Participants in this study reported an increasing likelihood of prescribing antibiotics as their shifts progressed, a finding that resonates with documented trends in observational studies by Linder and colleagues and Maier and colleagues, which also noted a rise in antibiotic prescriptions over time [15, 30]. Conversely, the trend regarding benzodiazepine prescriptions was less definitive; while participants in the current study raised benzodiazepines as a drug they were more likely to prescribe when decision fatigued, Maier and colleagues did not find conclusive quantitative evidence to support an increase [30]. Participants in the current study also reported a reduction in perceived confidence and an increased tendency to seek additional advice or confirmation from colleagues as shifts wore on. This broadly aligns with, and may go some way towards explaining, the finding that nurses become increasingly likely to refer patients on to other health professionals as time into shift increases [10]. It is important to highlight that there are currently no quantitative observational studies specifically addressing decision fatigue in the out‐of‐hours primary care setting. This gap suggests that trends observed in other contexts might manifest differently in this particular setting, warranting further investigation.

The study participants' self‐awareness of decision fatigue and their deliberate strategies to counteract its effects challenge previous assumptions that HCPs may not be conscious of this phenomenon [6]. HCPs seemed to know that their attention wanes over the course of the shift, leading them to potentially miss things or make inappropriate care decisions. This self‐awareness made some HCPs overly cautious, a phenomenon referred to by one participant as ‘looking for zebras instead of horses’, indicating an increased likelihood of overestimating the seriousness of common symptoms. This links in with Allan and colleagues's study of decision fatigue which found that nurses became more conservative in their triaging decisions over time [10], similar to the increased cautiousness described by this study's participants. The participants' self‐awareness of decision fatigue was not shared by all participants, as a minority stated that their decision‐making remains constant at all times. Statements about (professional) identity were also in conflict with this self‐awareness, as when participants spoke about their identity they described themselves as consistent in their decision‐making.

Environmental factors, such as workload and systemic issues, impacted decision‐making. For example, the pressure to manage long waiting lists was described as hindering decision consistency. While most decision fatigue studies strictly focus on completed workload and how the previously expended cognitive effort impacts future decision‐making, this finding aligns with research suggesting that unfinished workload (i.e., the work still to be done) also contributes to stress and decision fatigue [31].

Changes in emotion, as described in the fourth theme, fit with the extensive literature on compassion fatigue in healthcare providers [32]. Participants described a loss of empathy and an increased impatience towards their patients at later stages during their shifts. Our study also extends the compassion fatigue literature which currently focuses on a phenomenon that develops over extended periods and that is described as a lasting state. Our findings indicate that compassion fatigue can, like decision fatigue, take effect over shorter periods such as the course of a shift. Studies of multidisciplinary team meetings and decision fatigue have previously found that positive socio‐emotional interactions between health professionals decrease over time while negative interactions like disagreeing increase over time [33, 34, 35]. Our findings indicate that this might not only be the case for communication between colleagues but could also impact interactions with patients. We also found that social support from colleagues and positive patient interactions were said to bolster decision quality, suggesting that improvements in the work environment and support systems could mitigate decision fatigue.

The findings of this study contribute novel theoretical insights into understanding the phenomenon of decision fatigue. Traditionally, the decision fatigue literature has been dominated by the Strength/Resource Model of Self‐Control [36, 37] (commonly known as ego depletion) [12, 18, 22, 38, 39]. This framework posits that cognitive resources are finite and diminish with the exertion of self‐control. It suggests that continuous decision‐making tasks deplete these resources, leading to a decline in the capacity to make further effortful decisions. However, our study challenges this perspective by highlighting that changes in decision‐making may be more closely tied to fluctuations in motivation rather than resource depletion. Participants in our study reported noticeable temporal changes in motivation and mood and described using cognitive and behavioural strategies to uphold decision quality, which align better with process‐focused and opportunity cost models of fatigue [40, 41, 42]. Unlike depletion models which predict reliance on rest and breaks to restore depleted resources, participants in our study emphasised actively striving and employing structured approaches to sustain decision quality. While breaks were acknowledged as beneficial, the predominant use of motivational and cognitive strategies suggests that decision fatigue may stem not from an inability to exert effort but from a reduced willingness to do so. This interpretation resonates with quotes from participants that illustrate their decreasing motivation over the course of the shift and increasingly wanting to go home. This nuanced understanding underscores the potential to develop interventions focused on enhancing motivation and optimising decision‐making environments, rather than solely managing depleted cognitive resources.

It is essential to consider how training and systemic interventions could be designed to alleviate decision fatigue effects and enhance consistent patient care. While recommendations for specific interventions must be approached cautiously due to the current theoretical uncertainties surrounding the mechanisms that underly decision fatigue, raising awareness among HCPs about the concept of ‘decision fatigue’ could play a vital role in fostering a culture that normalises taking breaks as essential for maintaining quality care. Incorporating this awareness into training programs may help HCPs feel more supported in taking necessary breaks without fear of judgement. Additionally, appropriate staffing levels must be ensured so that HCPs have the resources to take the breaks they need. Participants in the present study frequently raised issues related to staffing, workload and the inability to take breaks as factors which exacerbated their decision fatigue. There is also a crucial need for experimental studies to be conducted in order to better understand the underlying mechanisms of decision fatigue. This will enable potentially effective interventions to be identified and tested.

## 5 Strengths and Limitations

This qualitative study is the first to explore the subjective experience of decision fatigue among HCPs. While the sample size was relatively modest, this represented excellent uptake, as *n* = 10 represents ~7% of the entire service bank workforce and ~29% of those regularly working shifts within the service. Thematic saturation was reached. Although the participant sample was drawn from a single geographical area, the sample's diversity, including GPs and ANPs with various levels of experience and shift frequencies, as well as those in leadership roles, suggests that the findings may be generalisable to the broader out‐of‐hours primary care clinician population in the UK. To gain a more comprehensive understanding of decision fatigue and further support the transferability of findings, future qualitative research should consider including diverse healthcare settings, regions, professional groups and patient groups where possible. This study's analysis employed an inductive approach, appropriate given the limited theoretical conceptualisation of decision fatigue in existing literature. Future studies could benefit from employing deductive approaches to further examine the theoretical frameworks suggested by these findings.

## 6 Conclusions

This study provides a comprehensive analysis of HCPs' experiences during lengthy shifts, revealing insights into their challenges, experiences and coping strategies regarding consistent clinical decision‐making. This research enhances understanding of the difficulties and mechanisms for managing decision‐making across extended work periods in healthcare and enables the advancement of the theoretical framework surrounding decision fatigue.

## Ethics Statement

This study involves human participants and was approved by the NHS Research Authority (East of England – Cambridge East Research Ethics Committee, 22/EE/0259).

## Consent

Participants gave informed consent to participate in the study before taking part.

## Conflicts of Interest

The authors declare no conflicts of interest.

## Data Availability

The dataset generated and anaysed during the current study is confidential due to the lack of participant consent for sharing and the risk of identification. Therefore, the data cannot be made publicly available.

## Endnotes

1 In medicine, the term ‘zebra’ is used to describe a rare disease or condition.

2 Escalating refers to the process of raising concerns or taking action when there are signs of patient deterioration or safety risks.

3 Diazepam belongs to a group of medicines called benzodiazepines. It is used to treat anxiety, muscle spasms and seizures or fits.
